# Risk factors for a decrease in high morale in very old people over a 5-year period: data from two Nordic countries

**DOI:** 10.1007/s10433-019-00521-1

**Published:** 2019-06-19

**Authors:** Marina Näsman, Johan Niklasson, Mikael Nygård, Birgitta Olofsson, Hugo Lövheim, Yngve Gustafson, Fredrica Nyqvist

**Affiliations:** 1 Social Policy Unit, Faculty of Education and Welfare Studies, Åbo Akademi University, PB311, 65101 Vaasa, Finland; 2 Department of Community Medicine and Rehabilitation, Geriatric Medicine, Sunderby Research Unit, Umeå University, 901 87 Umeå, Sweden; 3 Department of Community Medicine and Rehabilitation, Geriatric Medicine, Umeå University, 901 87 Umeå, Sweden; 4 Department of Nursing, Umeå University, 901 87 Umeå, Sweden

**Keywords:** Longitudinal studies, Aged 80 and over, Subjective well-being, Quality of life

## Abstract

High morale could be considered to be an essential part of aging well and increased knowledge of how to prevent a decrease in high morale in very old age could have important implications for policy, and social and health care development. The objective was to identify social and health-related risk factors for a decrease in morale over 5 years in very old people among those with high morale at baseline. The study is based on data derived from the Umeå85+/GERDA study conducted in Northern Sweden and Western Finland. The final sample consisted of 174 individuals who were 85 years and older at baseline and who had completed the follow-up 5 years later. Morale was measured with The Philadelphia Geriatric Center Morale Scale (PGCMS). A set of social and health-related variables were used to test which factors were associated with a decrease in morale over 5 years. Linear regression was used for the multivariable analyses. The sample had a mean change of − 1.3 (SD = 2.5) in PGCMS scores from T1 to T2. The results from the regression analyses showed that development of depressive disorders, increased feelings of loneliness and the death of a child during the follow-up period were associated with a decrease in morale. The results from our study indicate that preventing the development of depressive disorders and increasing loneliness are key factors in preventing a decrease in high morale. Additionally, very old people who have recently lost an adult child should receive adequate psychosocial support.

## Introduction

Population aging is a worldwide phenomenon and especially the amount of people aged 85 years and older are expected to increase rapidly (WHO 2011). As a consequence, active aging and successful aging (Foster and Walker 2015) have become key principles in public policy, and factors related to these concepts have been extensively studied among older people in general. Nevertheless, research focusing on aspects of active aging such as well-being explicitly in very old age is still limited. On the one hand, very old people tend to have a positive view on their health (French et al. 2012) and high levels of inner strength (Nygren et al. 2005), indicating that very old people can experience a high sense of well-being. On the other hand, very old age has often been associated with a high disease burden and different forms of losses, including decline in physical and cognitive function as well as social losses such as loss of spouse and friends (Baltes and Smith 2003). There are also studies showing that although the level of subjective well-being (SWB) seems to be stable from middle age to young old age, some dimensions of SWB decrease in very old age (e.g., Hansen and Slagsvold 2012), suggesting that very old people is a risk group requiring attention. Increased knowledge about SWB in very old age and its possible implications for social and health care is therefore essential. In this study, we analyze morale, seen as an aspect of SWB, by focusing on risk factors related to a decrease in high morale over 5 years in very old people.

Morale has been described as an overall sense of well-being, satisfaction with oneself, a certain acceptance of changes associated with aging (Lawton 1972, 1975), and future oriented optimism (Mannell and Dupuis 1996; McDowell 2006). Hence, morale can be seen as a dimension of subjective or psychological well-being, containing cognitive, social, and emotional aspects. High morale in very old people has been associated with increased 5-year survival (Niklasson et al. 2015a) and lower risk of depressive disorders over 5 years (Niklasson et al. 2017). It seems thus that high morale in very old age has some salutogenic features. At the same time, people with high morale are also more prone to have a decrease in morale over time, which make them interesting for a study focusing on changes (Näsman et al. 2019). By identifying risk factors for decreasing morale, preventive measures and interventions focusing on the main risk factors can be developed.

The vast majority of previous studies on morale in old age are cross-sectional, and most of them do not focus on very old age. In these studies, both social and health-related factors have been associated with lower morale. Social factors such as type of social network have previously been linked to morale in old age (e.g., Litwin 2001; Wenger et al. 1995). In Litwin’s (2001) study, individuals with more restricted social networks had lower morale than those with broad social networks that included family, friends and neighbors. Lower social support (de Guzman et al. 2015; Loke et al. 2011) and living alone (Iwasa et al. 2006) have further been associated with lower morale. In samples of very old people, lower satisfaction with support from family (Deng et al. 2010) and perceived loneliness (von Heideken Wågert et al. 2005) have been associated with lower morale. Hence, both more quantitative factors such as network composition and more qualitative social factors such as perceived loneliness seem to affect morale. Additionally, lower morale has been associated with sociodemographic factors such as lower level of education (Iwasa et al. 2006), lower income (Wenger et al. 1995), renting in opposite to owning one’s home (Breeze et al. 2004), and living in an institutional setting (von Heideken Wågert et al. 2005).

In the general elderly population, health-related factors such as fatigue, poor visual acuity, more non-prescriptive medicines for regular use (Mancini and Quinn 1981), chronic conditions (de Guzman et al. 2015; Iwasa et al. 2006), disability due to chronic illness (Loke et al. 2011), low levels of activity, problems with mobility, moderate to severe pain, and limitations in self-care (Kisely and Shannon 1999) have been associated with lower morale. Additionally, stroke (Niklasson et al. 2014) urinary tract infection (Eriksson et al. 2010), and cognitive function (Deng et al. 2010) have been associated with morale in very old age. Subjective assessments of one’s health also seem to affect morale. Both poorer self-rated health (Mancini and Quinn 1981; Wenger et al. 1995) and poorer comparative health (Mancini and Quinn 1981), i.e., lower assessment of one’s own current health status compared to 5 years ago, have been associated with lower morale. Regarding mental health, lower morale has been associated with psychological distress (Kisely and Shannon 1999), anxiety, and depression (Nagatomo et al. 1997). In very old age, both depression (Bergdahl et al. 2005) and depressive symptoms (von Heideken Wågert et al. 2005) have been associated with lower morale.

Further, it is evident that the relationship between health-related variables and morale in old age is complex, especially considering that different studies have come to somewhat conflicting results. Mancini and Quinn’s study (1981) found no associations between morale and for example the use of medical services or the number of illnesses, while the history of hospitalization in the study of Iwasa et al. (2006) and the number of chronic illnesses in the study of de Guzman et al. (2015) were found to be associated with lower morale. The differing results might partly be explained by the use of different measurements of illness and differences in methodology. To which extent health-related factors affect morale, especially over time, remains thus uncertain.

Some cross-sectional studies of morale have shown that the level of morale is higher in older ages (Iwasa et al. 2006; Woo et al. 2005), while others have shown that the level of morale is lower in advanced age (de Guzman et al. 2015). However, no study on morale in old age has, to our knowledge, focused on whether the same social and health-related factors affect morale in different age groups, i.e., if results regarding risk factors for lower morale in younger old also apply to very old age. Studies of other aspects of well-being in old age such as valuation of life indicate that health-related factors seem to be more important for younger old than in more advanced ages (Jopp et al. 2008). In support of this notion, Schöllgen et al. (2016) showed that the association between health and well-being (named health sensitivity by the authors) decreased with age, meaning that the weakest association was found in very old age. In contrast, social variables could be expected to have an important impact on well-being in very old age. For example, Pinquart and Sörensen (2000) found in their meta-analysis that both the quantity and the quality of social contacts were more important for SWB in older (mean age > 70 years) than in younger samples.

Further, very old people can according to Krause (2005) be more vulnerable to stress due to changes in physical and cognitive function. However, the results of his study indicated also that the stress-buffering effect of social support was most pronounced in the oldest old, meaning that they benefitted the most from emotional support. These findings could be supported by the social convoy model including stress, where the negative impact of stress, stemming from for example negative life events and daily hassles, is smaller for people with stronger social support and vice versa (Antonucci et al. 2009). Hence, it could be plausible that especially social risk factors, such as lack of social contacts and social support, play an important role in decreasing morale in very old age over time. Nonetheless, the relationship between age, social resources and SWB is not consistent. For example, there is a study showing that fewer social factors are related to SWB in very old age compared to younger old (Litwin and Stoeckel 2013), warranting further exploration. Litwin and Stoeckel (2013) also found that social networks matter in different ways in the two groups, for example that living with a spouse was associated with higher quality of life in younger old, while lower quality of life in older-old. In contrast, living with an adult child was associated with higher quality of life in older old and lower quality of life in the younger old.

Despite various results from cross-sectional studies, it is thus still unclear if these social and health-related risk factors also have an association with a decrease in morale over time, and in very old age. Especially to individuals aged 85 years and older, when changes in the life situation is likely to occur, studies of social and health-related changes in relation to changes in morale is of high relevance. Thus far, only a few studies (Klotz et al. 2018; Näsman et al. 2019; Scott and Butler 1997) have focused on decreasing morale and associated factors longitudinally. One of the studies focused on very old age, and included a sample with a mean age of 87 years at baseline (Näsman et al. 2019). In this study, negative life events seemed to have a cumulative negative effect on changes in morale over 5 years, i.e., the risk of a decrease in morale increased with the number of negative life events experienced. Further, the results showed that the two older age groups (90-year-olds and ≥ 95-year-olds) had a significantly higher score in the index of negative life events compared to the 85-year-olds, indicating that the risk of experiencing negative life events increased in advanced age. Another recent study focused on individuals in their fifties and in their seventies, and showed that worse oral health-related quality of life predicted lower morale over 10 years in both age cohorts (Klotz et al. 2018). A third study included individuals with a mean age of 71 at baseline (Scott and Butler 1997). In this 12-year follow-up study, marital status seemed to play an important role in decreasing morale. Being married at baseline was a significant predictor of lower morale after 12 years. Further, remaining married after 12 years was also associated with a decrease in morale, suggesting that being married, in opposite to being widowed or single, is contrary to what could be expected not always positively associated with well-being (see also Litwin and Stoeckel 2013). According to the authors, these results could have partly been connected to less social interaction and caregiving burden. Additionally, persons who had experienced a decrease in morale had to a larger extent also reported lower perceived economic adequacy. Interestingly, baseline variables included in their study explained only a small proportion of the variance in morale at follow-up. This suggests that variables regarding changes over the follow-up period could better explain changes in morale than baseline variables. However, considering the limited amount of longitudinal studies, as well as studies focusing on very old age, there is a need to further investigate morale in very old people over time.

The objective of this study is therefore to identify risk factors for a decrease in morale over 5 years in very old people among those with high morale at baseline. In order to examine this, we included social and health-related variables both describing the situation at baseline (T1) as well as corresponding variables reflecting changes that have occurred from baseline (T1) to follow-up (T2). In light of the study of Scott and Butler (1997), and that very old people are at high risk of experiencing social and health-related changes, we hypothesize that especially variables regarding changes over the follow-up period can explain a decrease in morale (Hypothesis 1). Furthermore, building upon previous cross-sectional research on morale and assumptions regarding characteristics of very old age, we expect that various social and health-related characteristics are important for explaining a decrease in morale over time. In particular, we hypothesize that social risk factors will play a prominent role in decreasing morale in very old age (Hypothesis 2).

## Methods

### Sample

The study is based on data from the Umeå85+/Gerontological Regional Database (GERDA), a population-based cohort study conducted in Northern Sweden and in Western Finland. Data have been collected in 2000–2002, 2005–2007, 2010–2012, and 2015–2017. In Sweden, the data have been collected in the city of Umeå and in five rural municipalities in the county of Västerbotten. Data were collected twice in Ostrobothnia in Finland, where two municipalities participated in 2005–2007 and four municipalities in 2010–2012. Participants were selected from the population register, acquired from the National Tax Board in Sweden and the Population Register Centre in Finland. Every second 85-year-old, every 90-year-old, and every ≥ 95-year-old were invited to participate.

Letters with information about the study were sent to individuals who were eligible to participate. These individuals were later contacted by phone in order to collect informed consent for participation in the study. In cases of cognitive impairment, informed consent was also collected from a next of kin. The data were collected through home visits, with a structured interview consisting of pre-defined questions and assessment scales. The interviewer was a physician, medical student, nurse or physiotherapist who had received education and training before conducting the interviews. In some cases, an additional interview was conducted with a next of kin and/or care staff for clarification.

To be included in the present study, the respondents needed to have completed a 5-year follow-up, with baseline (T1) data from 2000 to 2002, 2005 to 2007, or 2010 and follow-up (T2) data from 2005 to 2007, 2010 to 2012, or 2015. Further, having high morale (≥ 13 points in the Philadelphia Geriatric Center Morale Scale, PGCMS) at T1 and having answered 12 items or more in the PGCMS at both T1 and T2 (Niklasson et al. 2017; Näsman et al. 2019) were also criteria needed to be fulfilled for inclusion in the study.

**Fig. 1:**
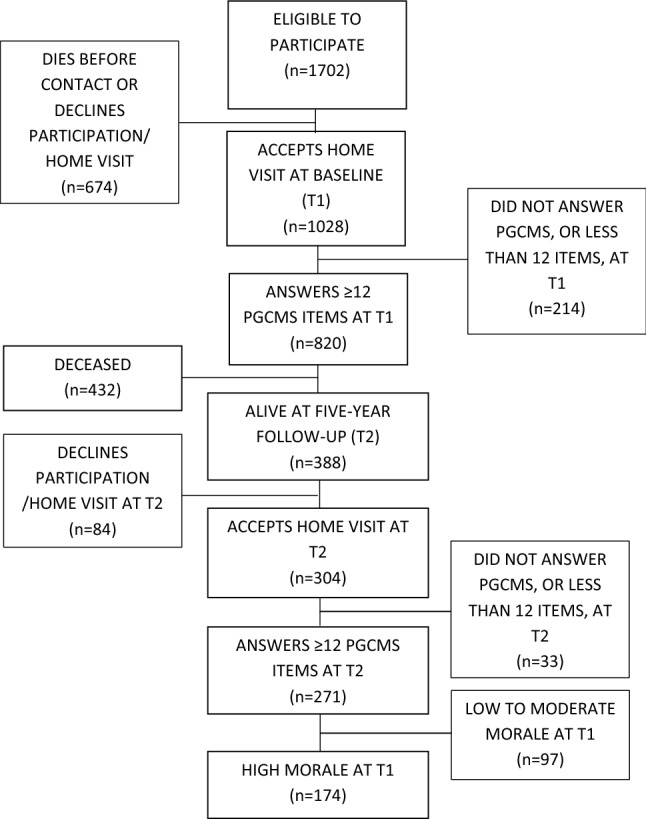
Flowchart describing the study population

Figure 1 gives an overview of how the study sample was selected. A total of 1702 individuals were eligible to participate at T1. Of them, 674 died before contact or declined to participate. Further, a part of the sample who accepted home visits at T1 did not answer PGCMS or less than 12 items (214/1028).

Of those who answered 12 items or more in PGCMS at T1, more than half died during the follow-up period (432/820). Those who died were significantly older (90.4, SD = 4.7 vs 87.4, SD = 3.4, *p* < .001), had significantly lower scores in Barthel’s index of activities of daily living (16.4, SD = 4.8 vs 19.1, SD = 2.4, *p* < .001), the Mini Mental State Examination (MMSE) measuring cognitive function (21.4, SD = 5.6 vs 25.4, SD = 3.5, *p* < .001), and PGCMS (11.3, SD = 3.1 vs 12.7, SD = 2.9, *p* < .001) compared to those who were alive at T2 (*n* = 388). Those who died had also to a higher extent depressive disorders at T1 (39.0% vs 23.3%, *p* < .001). There were no statistically significant differences between men and women nor regarding years of education.

Of those who were alive at T2, 84 individuals declined re-participation. Those who declined re-participation were to a statistically significant higher proportion women (82.1% vs 66.4%, *p *= .006), had significantly lower cognitive function according to MMSE (24.5, SD = 4.3 vs 25.6, SD = 3.2, *p *= .007), and had to a higher extent depressive disorders at T1 (34.5%, *n* = 29 vs 20.1%, *n* = 61, *p *= .005) compared to those who accepted home visits at T2 (*n* = 304). There were no statistically significant differences regarding age, years of education, PGCMS scores at T1 or activities of daily living.

Consequently, there were 304 individuals with sufficient PGCMS data at T1 who also accepted a home visit at T2, of whom 271 answered 12 items or more in PGCMS at T2. Of these, 97 individuals had low to moderate morale at T1 and were therefore excluded, leaving 174 individuals to be included in the analyses.

### Instruments

Morale was measured with The Philadelphia Geriatric Center Morale Scale (PGCMS), including the subscales agitation, lonely dissatisfaction, and attitudes toward own aging (Lawton 1975). The Swedish and the Finnish versions of the scale were translated from the British version of PGCMS (Challis and Knapp 1980), with 17 statements to which the respondent can chose between yes or no answers. Answers indicating high morale were given one point. In accordance with the scale instructions, zero points were given to answers indicating low morale and to unanswered questions (Lawton 2003). The maximum score of the instrument is thus 17 points. Scores ranging from 0 to 9 indicate low morale, 10–12 moderate morale, and 13–17 points high morale. The scale has been shown to have sufficient stability over time (Ma et al. 2010), although the factor structure of the subscales seemingly differ somewhat with increasing age (McCulloch 1991). Nevertheless, the psychometric properties of the Swedish version of the instrument have been tested and found satisfactory in a sample of very old people, with a Cronbach´s alpha of 0.74 indicating acceptable internal consistency (Niklasson et al. 2015b). As indicated in the introduction, and with regards to our second hypothesis, we concluded that both social and health-related variables ought to be examined when identifying risk factors for a decrease in morale. Next, the social and health-related variables included will be described in detail.

#### Social variables

The variable living alone was a dichotomous variable where persons who did not live with a partner or close relative were considered to live alone (living with someone = 0, living alone = 1). Individuals living in institutional care were also considered to live alone, since most rooms in nursing homes in both Sweden and Finland are single rooms. We chose to include the variable living alone instead of widowhood, as we regarded living alone as a more comprehensive measure including also cohabiting. The variable death of one’s child was included since adult children could be expected to be an essential part of the social network. Occurrence of the death of a child (no = 0, yes = 1) was assessed through self-report. Both living alone, in particular when a person starts to live alone during the follow-up period, and losing an adult child could indicate major social losses which could be expected to affect morale.

Since both quantitative and qualitative social aspects could be expected to affect morale, variables representing both were included. Quantity of social contacts was measured with two variables: numbers of visits received during a normal week and number of visits made by the respondent during the previous week. The variable describing number of visits received was dichotomized into two categories: 0–1 (coded as 1) and 2 or more visits (coded as 0). Visits from social and health care personnel were not included. The variable describing number of visits made by the respondent was also dichotomized (no visiting = 1, 1 visit or more = 0). These dichotomizations have also previously been used in other scientific articles based on the GERDA study (see for example Nyqvist et al. 2017). Feelings of loneliness were considered as a qualitative social factor and were measured with a dichotomized variable. Perceived loneliness was considered present if the respondent chose the answers “often” or “sometimes” as opposed to “seldom” or “never” to the question “Do you ever feel lonely?” (not lonely = 0, lonely = 1).

#### Health-related variables

A set of health-related variables, in which changes are likely to occur in very old age, were included as independent variables. Overall health status was assessed through self-report with the SF-12 item “In general, would you say your health is…” with the answer alternatives “excellent,” “very good,” “good,” “fair” and “poor,” The variable was dichotomized so that a respondent was considered to have poor self-rated health if he or she answered “fair” or “poor” (good = 0, poor = 1).

Activities of daily living (ADL) were assessed using the Barthel ADL index (Mahoney and Barthel 1965) with a maximum score of 20 points indicating total independence in personal ADL. The Mini Mental State Examination (MMSE) (Folstein et al. 1975) was used for assessing cognitive function. The instrument has a maximum score of 30 points, where higher scores indicate a higher level of cognitive function. Both instruments were used as continuous variables.

Two variables regarding sensory impairments were also included. A person was considered to have impaired hearing when he or she was unable to hear normal conversation at a 1 m distance with or without hearing aids (no impaired hearing = 0, impaired hearing = 1). Impaired vision was considered present if the individual was unable to read words written in 4-mm block letters with or without glasses (no impaired vision = 0, impaired vision = 1).

Depression, and depressive symptoms, has previously been associated with morale in several cross-sectional studies (Bergdahl et al. 2005; von Heideken Wågert et al. 2005; Nagatomo et al. 1997; Woo et al. 2005) and could also be regarded as an important mental health indicator to include considering the high prevalence of depressive disorders among very old people (e.g., Bergdahl et al. 2005). Depressive symptoms were screened for using the Geriatric Depression Scale with 15 items (GDS-15) (Sheikh and Yesavage 1986). If the interviewer was a physician or a specially trained medical student, the Montgomery-Åsberg Depression Rating Scale (MADRS) was also used (Montgomery and Åsberg 1979). Other psychiatric symptoms, as well as depressive symptoms, were also assessed using the Organic Brain Syndrome Scale (OBS) (Jensen et al. 1993). Depressive disorders were diagnosed according to the Diagnostic and Statistical Manual of Mental Disorders fourth edition (DSM-IV) (American Psychiatric Association 1994). The depressive disorders diagnosis was determined after reviewing medical records and the interview protocols including the instruments GDS-15, MADRS (if available), and OBS. Individuals with ongoing treatment for depressive disorders were also considered to have depressive disorders, regardless of the results from the assessment scales. The same specialist in geriatric medicine determined all medical diagnoses in the study during the whole study period in both Sweden and Finland.

### Analytical approach

The dependent variable was constructed to measure changes in PGCMS scores from T1 to T2, by subtracting the T1 scores from the T2 scores. A negative change is thus represented by a negative value in the variable. For example, having 17 points at T1 and 14 points at T2 would generate a value of − 3.

To test our first hypothesis, we included independent variables both from T1 and variables regarding changes from T1 to T2 in the analyses. As previously mentioned, these variables measured both social and health-related risk factors, in connection to our second hypothesis. To assess changes from T1 to T2 in the independent variables, continuous variables were computed by subtracting the T1 value from the T2 value. A negative value indicates thus a negative change. Regarding changes in dichotomous variables from T1 to T2, a negative change was given the value 1 while no change or improvement was given the value 0.

Given some inconsistencies in previous research regarding the association between age and morale, we also included age as a control variable in the multivariable analyses, to explore whether older age would be a risk factor of having a decrease in morale in a sample of very old adults (age range 85–99 years at T1). To further explore possible effects of age, interaction terms with age and the different social and health-related variables were constructed and tested.

Linear regression of the ordinary least square type (OLS) was used to test which of the social and health-related variables could predict a decrease in morale from T1 to T2. First, the independent variables were entered one by one, to test their association with changes in PGCMS scores (Model 0). Due to the relatively small sample size, a cutoff of *p* < .05 was chosen for the selection of variables in the multivariable regression models. In total, three multivariable models were tested. In Model 1, we tested if chosen variables from T1 could predict a decrease in morale from T1 to T2. In Model 2, we tested if chosen variables assessing changes from T1 to T2 could predict a decrease in morale. In the third model, variables from both Model 1 and Model 2 using the *p* < .05 criteria were included. All of the models also included age as a control variable. Models including gender as an additional control variable showed that gender had no effect on the results in this study and were therefore not presented. Based on the values of the variance inflation factors, there were seemingly no problems regarding multicollinearity between the independent variables. The IBM SPSS Statistics version 23 (IBM SPSS Inc., Chicago, IL, USA) was used for all calculations.

### Ethics

The study was approved by the Regional Ethical Review Board in Umeå (99-326, 05-063M, 09-178M, 14-221-31M, and 16-501-32M) and the Ethics Committee of Vaasa Central Hospital in Finland (05-87 and 10-54).

## Results

The sample (*n* = 174) had a mean age of 87.1 (SD = 3.1) at T1, and 60.9% (*n* = 106) were women. The sample had a mean of 7.3 (SD = 3.0) years of education and the majority, 89.1% (*n* = 155), were living in Sweden. At T1, the sample had a mean score in PGCMS of 14.7 (SD = 1.3) and the mean change from T1 to T2 was − 1.3 (SD = 2.5, range − 11 to 3) points. The majority of the sample (58.6%, *n* = 102) had a negative change of − 1 point or more, 33 individuals (19%) had no change, and 39 individuals (22.4%) had a positive change in PGCMS. Social and health-related characteristics at T1, as well as changes in the same characteristics from T1 to T2, are presented in Table 1.

**Table 1:** Sample characteristics at baseline (T1) and changes occurring from baseline (T1) to follow-up (T2) (*n* = 174)

Characteristics at T1	M(SD)/% (*n*)	Changes from T1 to T2	M(SD)/% (*n*)
PGCMS^a^	14.6 (1.3)	Changes in PGCMS scores^a^	− 1.3 (2.5)
*Social variables*			
Living alone^b^	64.9 (113)	Living with someone ➔ living alone^b^	11.5 (20)
Deceased children^b^	14.4 (25)	Child died during follow-up period^b^	6.3 (11)
0–1 visit received^b^	35.6 (62)	More than 1 visits received ➔ 0–1 visit received^b^	17.8 (31)
No visiting^b^	35.6 (62)	Visited more than once ➔ no visiting^b^	24.7 (43)
Feelings of loneliness^b^	29.9 (52)	No feelings of loneliness ➔ feelings of loneliness^b^	17.2 (30)
*Health-related variables*			
Barthel’s ADL index^a^ (0–20 points)	19.4 (1.9)	Changes in scores in Barthel’s ADL index^a^	− 2.2 (4.2)
MMSE^a^ (0–30 points)	26.0 (3.1)	Changes in MMSE scores^a^	− 3.7 (5.1)
Impaired hearing^b^	3.4 (6)	No impaired hearing ➔ impaired hearing^b^	16.7 (29)
Impaired vision^b^	4.0 (7)	No impaired vision ➔ impaired vision^b^	10.3 (18)
Poor self-rated health^b^	25.9 (45)	Good self-rated health ➔ poor self-rated health^b^	18.4 (32)
Depressive disorders^b^	7.5 (13)	No depressive disorder ➔ depressive disorder^b^	12.1 (21)

Mean and standard deviations are presented for continuous variables, and percentages and number of individuals for dichotomous variables

*PGCMS* the Philadelphia Geriatric Center Morale Scale, *MMSE* Mini Mental State Examination

^a^Continuous variables

^b^Dichotomous variables

The majority of the sample was living alone at T1 (64.9%), and 11.5% (*n* = 20) started to live alone during the follow-up period. There were 25 individuals (14.4%) who had a deceased child at T1, and from T1 to T2 an additional 6.3% (*n* = 11) had lost their child. As much as 29.9% (*n* = 52) experienced loneliness at T1, and during the follow-up period, 17.2% (*n* = 30) started to feel lonely. Regarding social contacts, 35.6% (*n* = 62) received 0–1 visits per week and 35.6% (*n* = 62) made no visits at T1. From T1 to T2, 17.8% (*n* = 31) of those who previously had more than one visit per week started to have one or less, and 24.7% (*n* = 43) of those who used to visit someone at least once a week started to make no visits (Table 1).

Regarding the health-related variables, the sample had a mean score of 19.4 (SD = 1.9) in the Barthel ADL index at T1 and had on average a decrease of − 2.2 (SD = 4.2) points from T1 to T2. In the MMSE, the sample had at T1 a mean score of 26.0 (SD = 3.1) and a mean decrease of − 3.7 (SD = 5.1) points from T1 to T2. Six individuals (3.4%) had impaired hearing at T1, and 16.7% (*n* = 29) got impaired hearing during the follow-up period. Regarding impaired vision, the corresponding numbers were 4.0% (*n* = 7) at T1, and 10.3% (*n* = 18) from T1 to T2. About a quarter of the sample (25.9%, *n* = 45) had poor self-rated health at T1, and 18.4% (*n* = 32) started to have poor self-rated health over 5 years. Lastly, 7.5% (*n* = 13) of the sample suffered from depressive disorders at T1, and 12.1% (*n* = 21) developed depressive disorders over the follow-up period.

Next, the bivariate associations between the characteristics at T1, as well as the changes from T1 to T2, and changes in PGCMS scores using linear regression (Model 0 in Table 2) were tested. Of the T1 variables, only poor self-rated health was significantly (*p *< .05) associated with a negative change in PGCMS scores over the follow-up period. Regarding the variables describing changes from T1 to T2, the social variables death of one’s child and increased feelings of loneliness were significantly associated with a negative change in PGCMS scores. Of the health-related variables, negative changes in ADL and MMSE, getting impaired vision, and the development of depressive disorders were associated with negative changes in PGCMS scores.

**Table 2:** Estimated effects of social and health-related variables at T1 and corresponding variables describing changes from T1 to T2 on changes in PGCMS scores (*n* = 174)

	Model 0		Model 1		Model 2		Model 3	
	*β*	*p*	*β*	*p*	*β*	*p*	*β*	*p*
*T1*								
Age (continuous)	− .022	.776	− .029	.700	.078	.328	− .029	.691
Social variables								
Living alone (no = 0, yes = 1)	.108	.156						
Deceased children (no = 0, yes = 1)	.012	.880						
0–1 visits received (> 1=0, 0–1 = 1)	− .097	.205						
No visiting (1 or more = 0, 0 = 1)	− .056	.472						
Feelings of loneliness (no = 0, yes = 1)	.057	.458						
Health-related variables								
Barthel’s ADL index (continuous)	− .007	.929						
MMSE (continuous)	.051	.500						
Impaired hearing (no = 0, yes = 1)	− .001	.987						
Impaired vision (no = 0, yes = 1)	− .126	.098						
Poor self-rated health (good = 0, poor = 1)	− .170	**.025**	− .171	**.025**			− .085	.254
Depressive disorders (no = 0, yes = 1)	− .120	.115						
*Changes from T1 to T2*								
Social variables								
Living with someone ➔ living alone (no = 0, yes = 1)	− .111	.143						
Child died during follow-up period ( no = 0, yes = 1)	− .175	**.022**			− .183	**.013**	− .194	**.009**
More than 1 visits received ➔ 0–1 visit received (no = 0, yes = 1)	.017	.828						
Visited more than once ➔ no visiting (no = 0, yes = 1)	− .049	.541						
No feelings of loneliness ➔ feelings of loneliness (no = 0, yes = 1)	− .228	**.003**			− .257	**.001**	− .229	**.002**
Health-related variables								
Changes in scores in the Barthel’s ADL index (continuous)	.241	**.001**			.071	.422		
Changes in MMSE scores (continuous)	.191	.**012**			.100	.268		
No impaired hearing ➔ impaired hearing (no = 0, yes = 1)	− .116	.130						
No impaired vision ➔ impaired vision (no = 0, yes = 1)	− .173	**.023**			− .137	.077		
Good self-rated health ➔ poor self-rated health (no = 0, yes = 1)	− .101	.192						
No depressive disorder ➔ depressive disorder (no = 0, yes = 1)	− .218	**.004**			− .174	**.022**	− .202	**.007**
Adjusted *R*^2^			.018		.157			.122

Standardized beta values are reported. Model 0 describes the bivariate association between each social and health-related variable and changes in PGCMS scores. Model 1 describes the association between T1 variables that were significant on a *p* < .05 level in Model 0, and changes in PGCMS scores. Model 2 describes the association between variables describing changes from T1 to T2 that were significant on a *p* < .05 level in Model 0, and changes in PGCMS scores. Model 3 includes variables that were significant on a *p* < .05 level in Model 1 and Model 2. Age is controlled for in Model 1, 2, and 3. Statistically significant results are emboldened  (*p* < .05)

*PGCMS* The Philadelphia Geriatric Center Morale Scale, *MMSE* Mini Mental State Examination

In Model 1, poor self-rated health at T1 remained statistically significant when controlling for age. In Model 2, recent death of one’s child, increased feelings of loneliness, and the development of depressive disorders remained statistically significant. In the final model (Model 3), all three variables selected from Model 2 (death of one’s child, increased loneliness, and development of depressive disorders) remained statistically significant, while poor self-rated health at T1 was no longer significant. We also tested for possible interaction effects with age and the different social and health-related variables, but no statistically significant interactions were found (not shown).

## Discussion

The objective of this study was to identify social and health-related risk factors for a decrease in morale over 5 years among very old people with high morale at baseline. The death of one’s child, development of depressive disorders, and increased feelings of loneliness from T1 to T2 remained statistically significant (*p* < .05) in the final regression model (Model 3 in Table 2). Of the T1 variables, only poor self-rated health was significantly associated with a decrease in morale, and lost statistical significance when variables regarding changes over the follow-up period were included in the same model. The results indicated thus that it is difficult to predict a decrease in morale over 5 years using baseline variables, likely due to that very old people are at high risk of experiencing social and health-related changes over a relatively short time period. Hence, the results corroborate our first hypothesis that the use of variables regarding changes over the follow-up period would be important when identifying risk factors for a decrease in high morale. The results partly support our second hypothesis that social risk factors, in this case loss of child and perceived loneliness, would play a prominent role in decreasing morale in very old age. In this sample, we found no significant interaction effects between age and the social and health-related variables, indicating that the risk factors had similar effects on morale regardless of age.

Regarding the social variables, one could conclude from Table 1 that there is an increase in the occurrence of potential risk factors for having a decrease in morale. For example, the number of people living alone increased, and the number of social contacts decreased. In this study, however, perceived loneliness emerging over the follow-up period and the recent loss of a child were the only social factors significantly associated with a decrease in high morale. Perceived loneliness is common in very old age (Nyqvist et al. 2017), has previously been associated with lower morale cross-sectionally (von Heideken Wågert et al. 2005), and is associated with many other negative outcomes (see for example Luanaigh and Lawlor 2008). Importantly, depression and loneliness in old age are often highly correlated (see for example Cacioppo et al. 2006). However, the results of our study showed that both depression and loneliness remained statistically significant in the regression models which corroborate the notion that loneliness and depression should be considered separate constructs (Cacioppo et al. 2006). Nevertheless, interventions targeting loneliness could also have an effect on depressive symptomatology and vice versa (Cacioppo and Hawkley 2009). Considering that living alone and living in institutional settings are major risk factors for experiencing loneliness in very old age (Nyqvist et al. 2017), actions preventing loneliness could especially be targeted at these two groups.

Our results concerning that recently losing a child is associated with decreasing morale supports the notion that adult children are an essential part of the social network in very old age (Litwin and Stoeckel 2013). The result is also in line with previous studies on bereavement in very old age, which have indicated that bereavement has a great impact on psychological dimensions of health (d’Epinay et al. 2010). Losing an adult child has also been associated with depression in very old age (Bergdahl et al. 2005). Hence, it is plausible that losing a child, depressive symptoms and feelings of loneliness are interrelated. However, research focusing on losing a child in very old age seems limited, implying that more research on this topic is needed. Additionally, the knowledge and tools required for managing bereavement in social and health care targeting older people are lacking, warranting increased attention and development (Morris et al. 2018; Van Humbeeck et al. 2016).

As seen in Table 1, there is a notable increase from T1 to T2 in all health-related risk factors included in this study. Even though many health-related variables have previously been associated with lower morale in cross-sectional studies, development of depressive disorders was the only variable remaining statistically significant in the multivariable analyses in our study. It seems thus that health status predicts to a lesser extent morale over time in very old people, which would be in line with the notion regarding health-sensitivity, i.e., that the association between health and perceived well-being weakens with increasing age, as suggested by Schöllgen et al. (2016). Nevertheless, our results suggest that mental health is crucial to well-being in this age group and that depression and depressive symptoms are not only associated with lower morale cross-sectionally (Bergdahl et al. 2005; von Heideken Wågert et al. 2005; Nagatomo et al. 1997) but also longitudinally. Considering the many other known negative effects, such as higher mortality risk (Bergdahl et al. 2005), of depressive disorders in very old age, preventing depressive disorders is utmost important. Although knowledge regarding mental health care interventions targeting older people is limited, a recent review by Biering (2018) reports promising results when using holistic treatment models focusing on both health-related and psychosocial well-being. A holistic model of social and health care could potentially cover both the prevention of loneliness and depression, as well as support to those grieving the loss of a child, and should therefore be further developed and tested to prevent decreasing morale among very old people.

The study is based on a representative sample of very old people aged 85, 90, and ≥ 95 at T1 in Northern Sweden and Western Finland including individuals both living in their own homes and in institutional settings. The longitudinal design of the study also adds valuable information to the field. However, there are some potential limitations that should be addressed. First, a high proportion of the sample died during the follow-up period (see Fig. 1). Even though this could be expected when assessing very old people, it affects the generalizability of the results. Those who died were significantly older, had to a higher extent depressive disorders, and had lower scores on MMSE, Barthel’s ADL index and PGCMS compared to those who were alive at T2. There were also individuals who declined participation at T2. The individuals who declined participation at T2 were to a higher proportion women, were assessed with lower cognitive function, and had to a higher extent depressive disorders than those who accepted re-participation. Second, the length of the follow-up period could have affected the results. For example, the indication that it was difficult to predict a decrease in morale based on baseline variables could partly be due to the length of the follow-up period, where a shorter follow-up period might have generated different results. Third, there is a possibility that factors not included in this study, such as factors related to economy (Scott and Butler 1997; Wenger et al. 1995) or personality (Loke et al. 2011), also might influence morale over time. This information was, however, not available in our data. Future studies of morale in very old age could also attempt to identify factors associated with an increase in morale, which could provide essential information on how to promote high morale.

### Implications

High morale seems to play an important part in the process of aging, and it is therefore urgent that preventive measures against decreasing morale are developed in social and health care targeting very old people. Based on the results from this study, it seems difficult to predict a decrease in high morale over 5 years among very old people. Rather, increases in depressive symptomatology and loneliness in very old age over time should receive adequate attention in order to prevent decreasing morale. Additionally, appropriate psychosocial support should be given to those who have recently lost an adult child.
